# Research progress of flexible pressure sensor based on MXene materials

**DOI:** 10.1039/d3ra07772a

**Published:** 2024-03-21

**Authors:** Zhigang Hu, Feihu Xie, Yangyang Yan, Hanjing Lu, Ji Cheng, Xiaoran Liu, Jinghua Li

**Affiliations:** a College of Medical Technology and Engineering, The 1st Affiliated Hospital, Henan University of Science and Technology Luoyang 471000 China anubiss1860@163.com; b Key Laboratory of Hainan Trauma and Disaster Rescue, The 1st Affiliated Hospital, College of Emergency and Trauma, Hainan Medical University Haikou 570100 China; c Luoyang Ship Material Research Institute, China Shipbuilding Industry 725 Research Institute Luoyang 471000 China

## Abstract

Flexible pressure sensors overcome the limitations of traditional rigid sensors on the surface of the measured object, demonstrating broad application prospects in fields such as sports health and vital sign monitoring due to their excellent flexibility and comfort in contact with the body. MXene, as a two-dimensional material, possesses excellent conductivity and abundant surface functional groups. Simultaneously, MXene's unique layered structure and large specific surface area offer a wealth of possibilities for preparing sensing elements in combination with other materials. This article reviews the preparation methods of MXene materials and their performance indicators as sensing elements, discusses the controllable preparation methods of MXene materials and the impact of their physical and chemical properties on their functions, elaborates on the pressure sensing mechanism and evaluation mechanism of MXene materials. Starting from the four specific application directions: aerogel/hydrogel, ink printing, thin film/electronic skin, and fiber fabric, we introduce the research progress of MXene flexible pressure sensors from an overall perspective. Finally, a summary and outlook for developing MXene flexible pressure sensors are provided.

## Abstract 2

This manuscript summarizes the preparations and potential applications of MXene-associated technology and provides a comprehensive review of the recent development of high-performance flexible pressure sensors based on MXenes materials.

## Graphical abstract

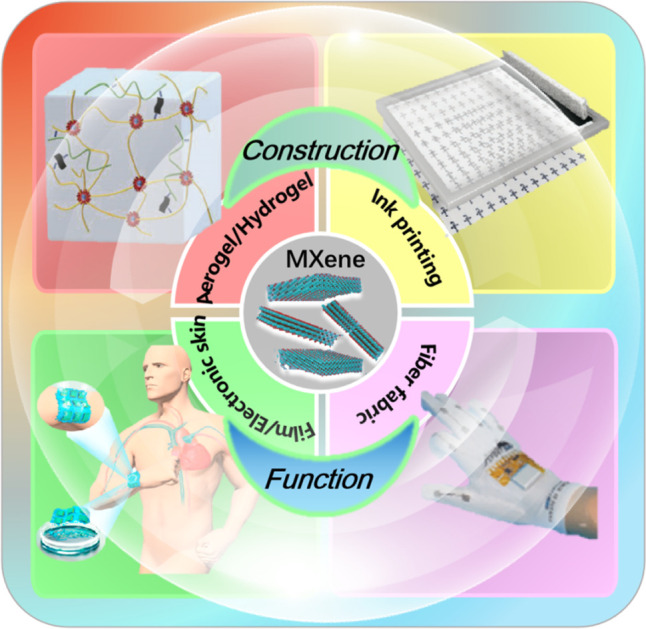

## 1 Introduction

With the rapid advancement of flexible electronic technology, flexible sensors have garnered considerable attention in various domains, including human health monitoring, motion monitoring, intelligent dressing, and human–computer interaction.^1–4^ characterized by their exceptional flexibility, high sensitivity, and lightweight, flexible sensors find extensive applications in the measurement of diverse human body information such as joint movement, sweat, and temperature. Sensors can be categorized into pressure sensors, temperature sensors, gas sensors, and biosensors based on their specific measurement capabilities.^5–8^ Regardless of their type, sensors fundamentally convert non-electrical quantities into electrical signals, thus enabling the measurement, transmission, processing, and control of different signals. In the case of pressure sensors, they can be further classified into piezoresistive, capacitive, and piezoelectric types depending on their underlying sensing mechanisms.^9–11^ In recent years, various nanomaterials such as graphene, carbon nanotubes, metal nanowires, carbon black, MXene, and various MOFs have been used as sensing materials to develop flexible pressure sensors with excellent sensitivity.^12–19^

**Fig. 1:**
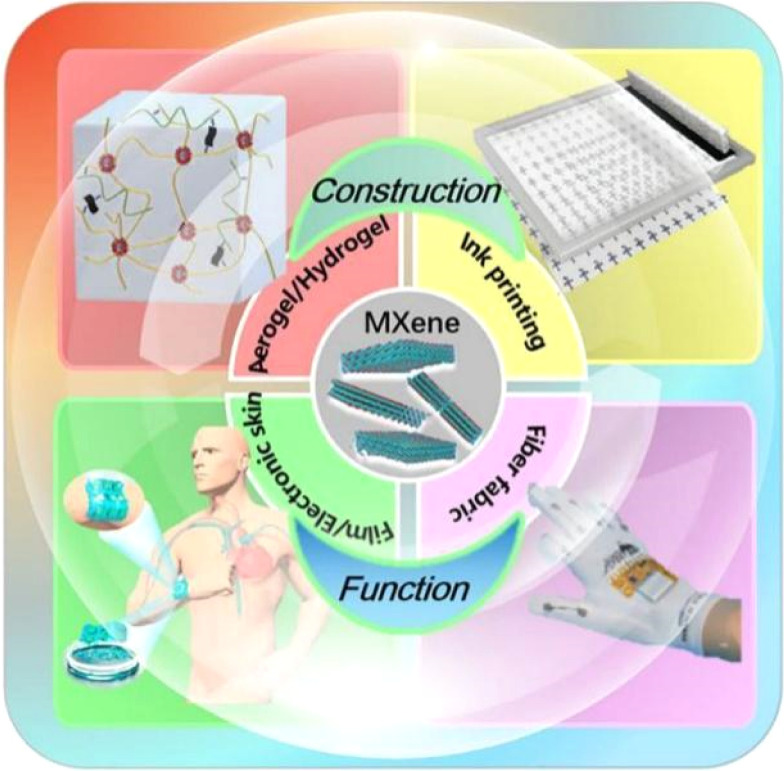
Sensing approaches for flexible sensors based on MXene: aerogel/hydrogel, ink printing, film/electronic skin, and fabric.

MXene is a type of two-dimensional layered nanomaterial characterized by its general chemical formula M_*n*+1_X_*n*_T_*x*_ (*n* = 1, 2, 3), where M represents transition metals (such as Sc, Ti, Zr, V, Nb, *etc.*), X represents carbon and/or nitrogen, and T_*x*_ represents surface functional groups (such as –OH, =O, –F, *etc.*). Among the reported sensing materials, MXene stands out for its large specific surface area and abundant functional groups on the surface. MXene has metal-like electrical conductivity, and its abundant functional groups on the surface (–OH, =O, –F, *etc.*) give it good hydrophilicity and easy to form micro–nano-structures by chemical bonding with other materials. In terms of mechanical properties MXene materials have excellent flexibility and stretchability, which enables MXene materials to adapt to a wide range of complex strain scenarios such as bending, stretching, or compression for reliable mechanical sensing. After continuous research, the tensile and compressive properties of flexible pressure sensors have been further improved by changing the structure of the sensing layer material, such as bionic structure, island bridge structure, *etc.*^20,21^ However, it remains challenging for a single material to simultaneously meet the requirements for flexibility, electrical conductivity, biocompatibility, and resistance to magnetic interference which are crucial for diverse applications. Composite materials offer a solution by overcoming the limitations of individual materials and can yield flexible sensors with superior performance. Zhu *et al.*^22^ prepared MXene/bamboo fiber (BF) composite paper with excellent flexibility and mechanical properties by vacuum filtration. Due to the excellent mechanical properties of MXene and the formation of strong bonds with bamboo fibers, the composite paper exhibits an excellent tensile strength of 49.5 MPa, which is higher than that of the MXene sheet and the bamboo fiber film alone. Cheng *et al.*^23^ proposed a fast gas-foaming strategy to construct MXene aerogels with tunable interlayer spacing. The presence of MXene provides high conduction efficiency and maximizes electron channels under pressure, which is conducive to the effective utilization of the metallic properties of the MXene surface. The aerogel can generate maximized electron channels under pressure, which is conducive to the effective utilization of the metallic properties of the MXene surface. The adjustable interlayer structure of MXene, the rich functional groups on the surface, and the excellent compatibility of MXene give it a better adaptability and performance advantage in compositing with other materials. This paper provides a comprehensive review of the recent research progress made in utilizing MXene and other materials for the development of high-performance flexible pressure sensors (Fig. 1). Specifically, the review focuses on the perspectives of aerogel/hydrogel, ink printing, film/electronic skin, and fiber fabric.^24–27^

## 2 Preparation and properties of MXene materials

### 2.1 Preparation of MXene

In 2011, Gogotsi *et al.* performed the first selective etching of aluminum (Al) from aluminum titanium carbide (Ti_3_AlC_2_) using hydrofluoric acid (HF). This process allowed for the preparation of multi-layer MXene powder Ti_3_C_2_T_*x*_.^28^ Subsequently, researchers have synthesized various MXene materials such as Ti_2_CT_*x*_, V_2_CT_*x*_, Cr_2_CT_*x*_, Zr_2_CT_*x*_, *etc.*, expanding the family of MXene to include transition metal carbides, nitrides, and carbon nitrides.^29^ The chemical equation for etching titanium-carbide aluminum with hydrofluoric acid is as follows:1
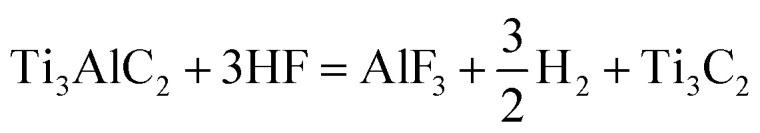

2 Ti_3_C_2_ + 2H_2_O = Ti_3_C_2_(OH)_2_ + H_2_

3 Ti_3_C_2_ + 2HF = Ti_3_C_2_F_2_ + H_2_

**Fig. 2:**
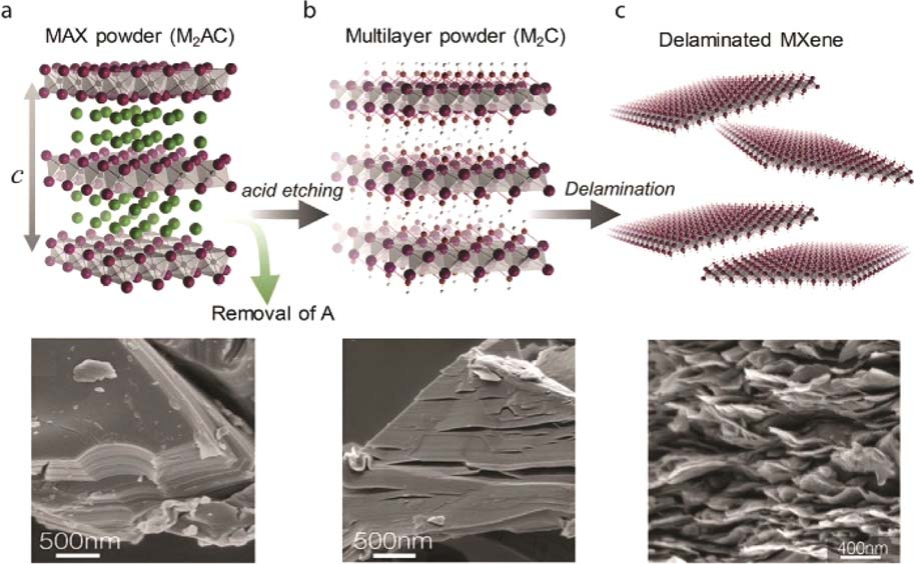
Pathways to synthesize MXene flakes using a top-down etching method. Copyright© 2020, John Wiley and Sons.

The layered MXene material with an organized structure was obtained by stratifying titanium-carbide aluminum through etching with hydrofluoric acid. However, as hydrofluoric acid is corrosive to organisms, Ghidiu *et al.*^30^ employed the LiF/HCl mixture etching method to produce MXene. This resulted in Ti_3_C_2_T_*x*_ with clay-like morphology characteristics, which could be further separated into single pieces of Ti_3_C_2_T_*x*_ with sub-micron transverse size by ultrasonic treatment in water. Additionally, it has been reported that MXene can be efficiently stratified using tetrabutylammonium hydroxide (TBAOH) and an amine-assisted method (Fig. 2), which helps improve the yield of MXene material.^31,32^ At present, MXene materials have colloidal solution, powder, self-supporting membrane, and other forms, now meet the general preparation methods of flexible sensors include coating, printing, wet spinning, vacuum filtration, *etc.*, a variety of forms for the preparation of subsequent sensing materials provide more possibilities.^33–36^

### 2.2 Electrical property

**Fig. 3:**
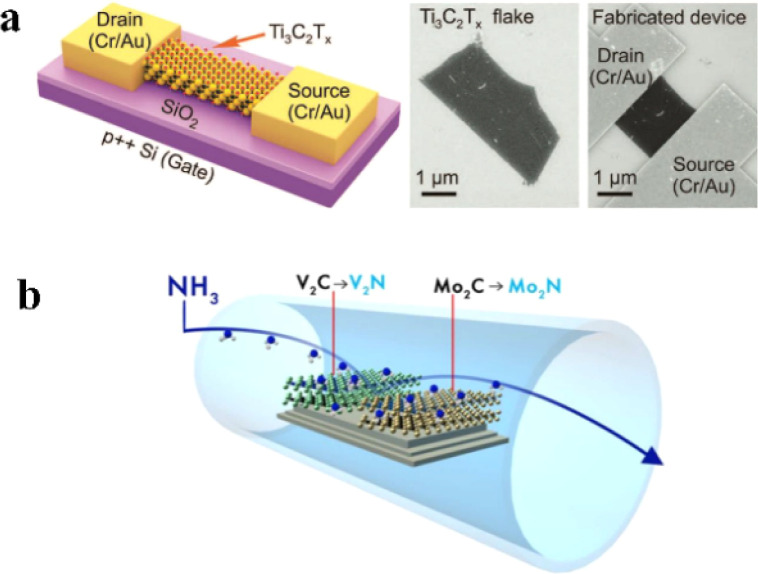
(a) Schematic and SEM images of Ti_3_C_2_T_*x*_-based field effect tubes. Copyright© 2016, John Wiley and Sons. (b) Schematic representation of 2D transition metal nitrides by elevated temperature ammoniation. Copyright© 2016, Royal Society of Chemistry.

The conductivity of the sensing material is a key factor affecting the sensing performance of flexible sensors, and MXene has a high conductivity similar to that of metals. Lipatov *et al.*^37^ prepared field-effect transistors (FETs) based on a single Ti_3_C_2_T_*x*_ sheet as shown in Fig. 3a and measured their electrical properties. The single Ti_3_C_2_T_*x*_ sheet exhibited a high conductivity of 4600 ± 1100 S cm^−1^ and field-effect electron mobility of 2.6 ± 0.7 cm^2^ V^−1^ s^−1^.

According to density-functional theory (DFT), the electrical properties of MXene are related to its elemental composition and surface groups.^38^ Targeted control of the electrical properties can therefore be achieved by purposefully changing the elemental composition and/or surface terminations of MXene. For example, in Urbankowski's work,^39^ Mo_2_C was used as a carbide MXene precursor, which was converted to Mo_2_N phase by ammonia decomposition (Fig. 3b). After heat treatment at 600 °C, Mo_2_N retained the layered structure of MXene. During subsequent electrical resistance measurements, the pristine Mo_2_CT_*x*_ films were treated under the same conditions in an inert atmosphere to eliminate defects introduced by the heat treatment. The results showed that the resistivity of the treated Mo_2_CT_*x*_ films was still an order of magnitude higher than that of the Mo_2_NT_*x*_ films.

In addition, the actual electrical properties of MXene films are macroscopic manifestations of multilayer stacked nanosheets, and thus the intercalation between the layers also determines their electrical properties. For example, cations in reagents (tetramethylammonium ions (TMA^+^), ammonium ions (NH_4_^+^), and lithium ions (Li^+^)) and organic molecules (dimethylsulfoxide (DMSO) and isopropylamine) can be inserted into the spacing of MXene's layers, altering the electrical properties of the MXene films.^40,41^ Therefore, the elemental composition, surface termination, and embedding of MXene films can be controlled by post-processing modifications to effectively achieve targeted control of their electrical properties.

It is worth noting that MXene's large specific surface area and surface-rich functional groups combine with H_2_O, O_2_, and free ions in the air when exposed to air, which inhibits the transfer of interlayer electrons and weakens the electrical conductivity of MXene, and thus the storage environment and the storage time can seriously affect the electrical conductivity of MXene materials.

### 2.3 Mechanical property

The mechanical properties of flexible sensing materials play a crucial role in determining the flexibility, detection range, and durability of flexible pressure sensors, which are important parameters for evaluating flexible pressure sensing materials. Guo *et al.*^42^ investigated the mechanical properties of monolayer MXene using first-principles calculations, taking Ti_*n*+1_C_*n*_ as an example. Experimental results demonstrate that two-dimensional Ti_2_C can endure significant strains of 9.5%, 18%, and 17% under biaxial and uniaxial tension along the *x*/*y* directions, respectively. After modifying the oxygen through surface functionalization, the strain of Ti_2_CO_2_ increased to 20%, 28%, and 26.5% respectively (exceeding the biaxial strain limit of 15% for graphene).

**Fig. 4:**
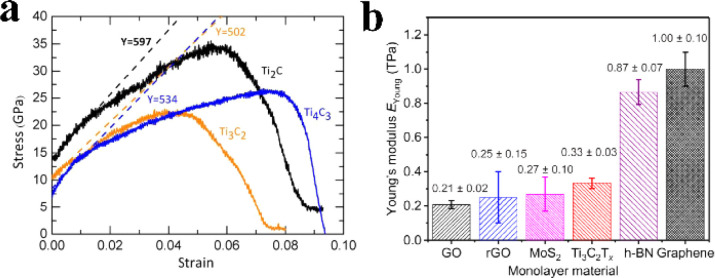
Mechanical properties of MXene materials (a) strain–stress curves of Ti_*n*+1_C_*n*_ samples during tensile loading. (b) Young's modulus of several 2D materials. Copyright© 2018, AAAS.

Borysiuk *et al.*^43^ constructed an ideal Ti_*n*+1_C_*n*_ monolayer model and performed classical molecular dynamics simulations to obtain the stress/strain curve of the MXene sample under tensile load, as shown in Fig. 4a. For a strain *ε* < 0.01, the Young's modulus values of Ti_2_C, Ti_3_C_2_, and Ti_4_C_3_ are 597 GPa, 502 GPa, and 534 GPa, respectively. In addition to computational studies, Lipatov *et al.*^44^ conducted atomic force microscopy (AFM) nanoindentation experiments to measure the mechanical properties of MXene. They utilized a tip with a radius of 7 nm and obtained the force–displacement curve. The Young's modulus of single-layer Ti_3_C_2_T_*x*_ was determined to be 0.33 ± 0.03 TPa (Fig. 4b). Therefore, MXene is an excellent choice for applications requiring nanodevices and composites with high mechanical property demands. Although monolayer MXene exhibits a broad theoretical strain range and high Young's modulus, in pressure sensor applications, MXene is typically composed of laminated nanosheets. This introduces certain disparities between the theoretical and actual mechanical properties. In the presence of an external load, cracks tend to emerge between the nanosheets rather than within the sheets themselves, as the binding forces between the nanosheets are predominantly governed by van der Waals interactions. Strengthening these interface interactions becomes essential. Consequently, during the fabrication of MXene sensing materials, various compounds such as polyvinyl alcohol (PVA), cellulose nanofibers (CNFs), and polyaniline (PANI) are employed to enhance the bonding strength between the nanosheets. This effective approach significantly enhances the mechanical properties of the sensing materials.^45^

## 3 Pressure sensing mechanism and evaluation mechanism of MXene material

### 3.1 Sensing mechanism

#### 3.1.1 Piezoresistive pressure sensor

**Fig. 5:**
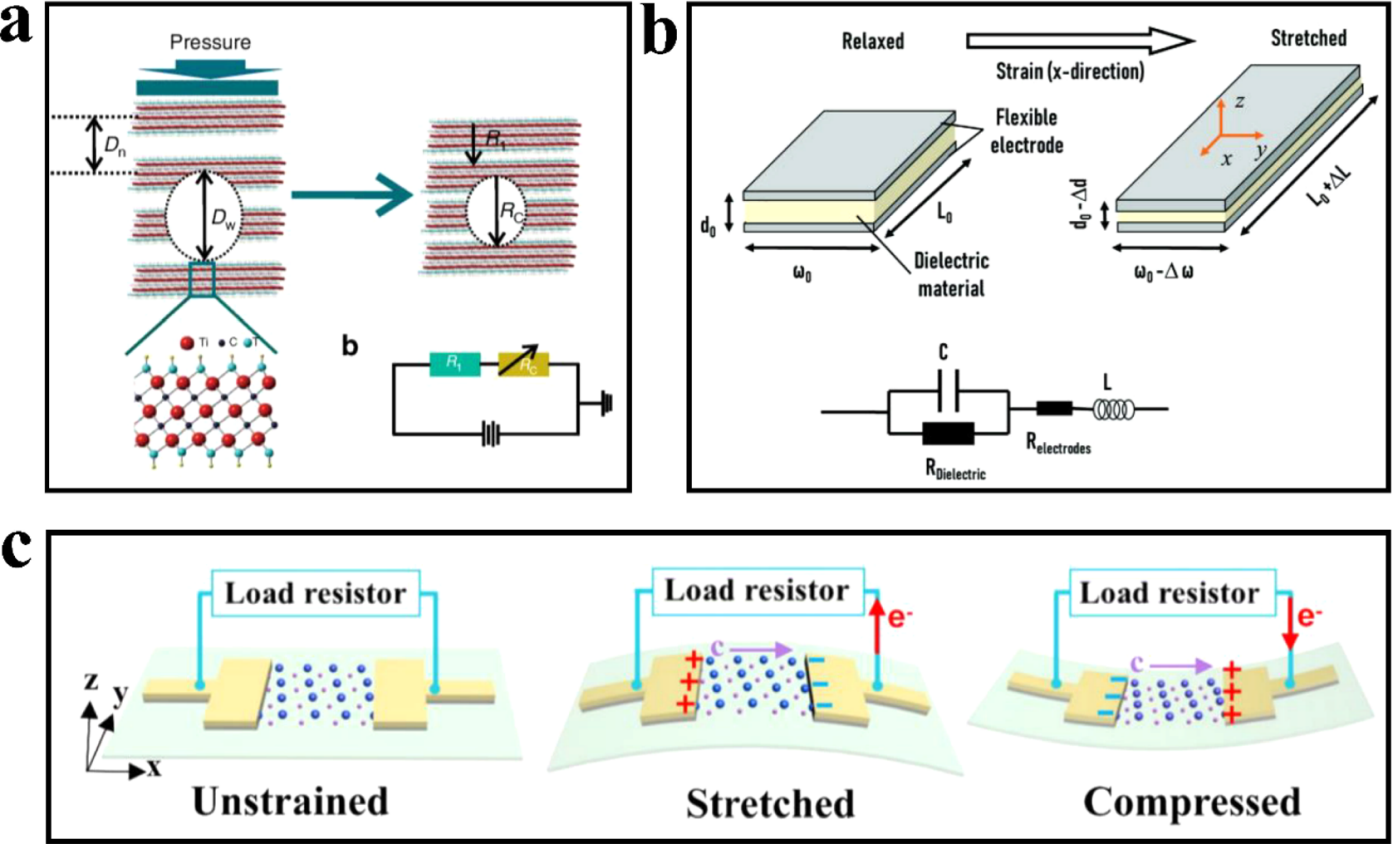
(a) The working mechanism of piezoresistive sensor based on MXene material. Copyright© 2017, Springer Nature. (b) Schematic diagram of flexible capacitive sensor. Copyright© 2021, John Wiley and Sons. (c) Working mechanism of a single layer Ti_3_C_2_T_x_ MXene piezoelectric sensor Copyright© 2021, Elsevier.

When an external load is applied to the piezoresistive sensor, the sensing material undergoes deformation, leading to a change in the conductive path within the material. This change is then manifested as a variation in resistance that can be measured externally. The working mechanism of the piezoresistive sensor based on MXene material is depicted in Fig. 4. In this figure, *R*_total_ represents the total resistance, while *R*_1_ corresponds to the resistance of MXene nanosheets with initially small spacing. Under pressure, the resistance of *R*_1_ remains nearly constant. On the other hand, *R*_c_ represents the resistance between MXene sheets with initially larger spacing, which decreases as the distance between adjacent MXene sheets reduces due to the external load (Fig. 5a). Consequently, the internal resistance decreases, resulting in an overall increase in conductivity. The larger the distance *D*_w_ between the two lamellae, the easier it is compressed, and the smaller the distance *D*_n_ between the two lattices, the smaller the compression ratio.^46^

#### 3.1.2 Capacitive pressure sensor

Capacitive pressure sensors detect external pressure by monitoring the change in capacitance when subjected to mechanical pressure. The sensing mechanism of a capacitive sensor is illustrated in the figure.^47^ To optimize the performance of capacitive sensors, various parameters are typically adjusted, such as the electrode area, the distance between the electrodes on both sides, and the dielectric constant of the material (Fig. 5b). The dielectric layer is sandwiched between two flexible electrodes, and the size of the sensor changes when subjected to external forces. *C* represents the central component of the capacitor; *R*_electrodes_ represent the residual resistance of the electrode; *R*_dielectric_ represents the current leaking in the dielectric material; *L* is the inductance of the wire. MXene materials, owing to their high conductivity and flexibility, can serve as electrodes in capacitive sensors or be utilized to fabricate microstructures that enable variable area modulation under pressure.^48^

#### 3.1.3 Piezoelectric sensor

The piezoelectric pressure sensor is primarily based on the characteristics of piezoelectric materials. Under the influence of external force, the material's internal charge becomes non-uniform, resulting in charge polarization. The charge polarization is eliminated upon external unloading. Studies have demonstrated that MXene exhibits a high orientation as a piezoelectric material with a non-centrosymmetric lattice structure. Fig. 5c presents a schematic representation of a single-layer Ti_3_C_2_T_*x*_ piezoelectric device. This device achieves tensile and compressive strain of Ti_3_C_2_T_*x*_ by bending the PET substrate outward and inward. The electrodes of the device are connected to an external circuit for the measurement and recording of piezoelectric signals. The red and purple arrows represent the current direction and polarity direction of Ti_3_C_2_T_*x*_ under different states, respectively.^49^

### 3.2 Evaluation index

#### 3.2.1 Sensitivity

The sensitivity of a flexible sensor is the degree to which the sensor responds to a change in an external physical quantity. In flexible sensors, sensitivity usually refers to the proportionality of the sensor's output signal relative to the change in the input signal. The sensitivity of a flexible sensor is usually measured by its GF (Gauge factor), and in general, a larger GF value indicates a higher sensitivity of the sensor and a more sensitive response to external physical quantities. In previous reports, the emergence of nonlinear sensors *i.e.*, sensors with varying sensitivity under different sizes of pressure operating ranges, which is related to the sensing material as well as the sensor structural design, can be realized through the selection of appropriate GF values for accurate monitoring and control of different application scenarios.

#### 3.2.2 Detection range

The detection range refers to the load range within which the sensor can reliably detect changes in electrical signals under normal working conditions. In practical sensor monitoring, selecting a sensor with high sensitivity within the appropriate detection range enables more accurate measurement. Conversely, operating the sensor outside the detection range can shorten its service life or cause damage.

#### 3.2.3 Linearity

Linearity refers to the degree of linearity in the actual relationship curve between the sensor input and output. Under specified conditions, linearity is determined by the maximum deviation between the sensor's calibration curve and the fitted line, expressed as a percentage of the full-scale output. A smaller value indicates better linear characteristics, resulting in more accurate detection signals and facilitating subsequent signal processing. This allows for direct use in calibration, display, or control using simple algorithms or circuits.

#### 3.2.4 Response time

The response time is an indicator of the speed at which the electrical signal of the strain sensor changes when it is subjected to a load. The main factors that influence the response time include the viscoelasticity of the sensing material and the stability of both the sensing material and the electrode. Typically, the response time ranges from tens of milliseconds to hundreds of milliseconds. However, in piezoresistive sensors, response time is longer compared to capacitive sensors due to the influence of the internal conductive structure.

The performance of the sensor can be evaluated based on several factors. Apart from the aforementioned sensor evaluation indicators, flexibility, hysteresis, accuracy, and durability are also crucial indicators for assessing sensor performance.

## 4 Research progress of pressure sensor based on MXene material

### 4.1 Aerogel/hydrogel

Aerogel is a nanoscale porous solid material formed by the sol–gel method. This method replaces the liquid phase in the gel with gas through a specific drying process. With its three-dimensional network structure filled with gas, aerogel exhibits high porosity, high specific surface area, low density, and low thermal conductivity. In recent years, researchers have incorporated various nanoparticles, conductive polymers, and carbon materials into the aerogel matrix, leveraging nanotechnology to produce composite aerogels for sensing devices.

**Fig. 6:**
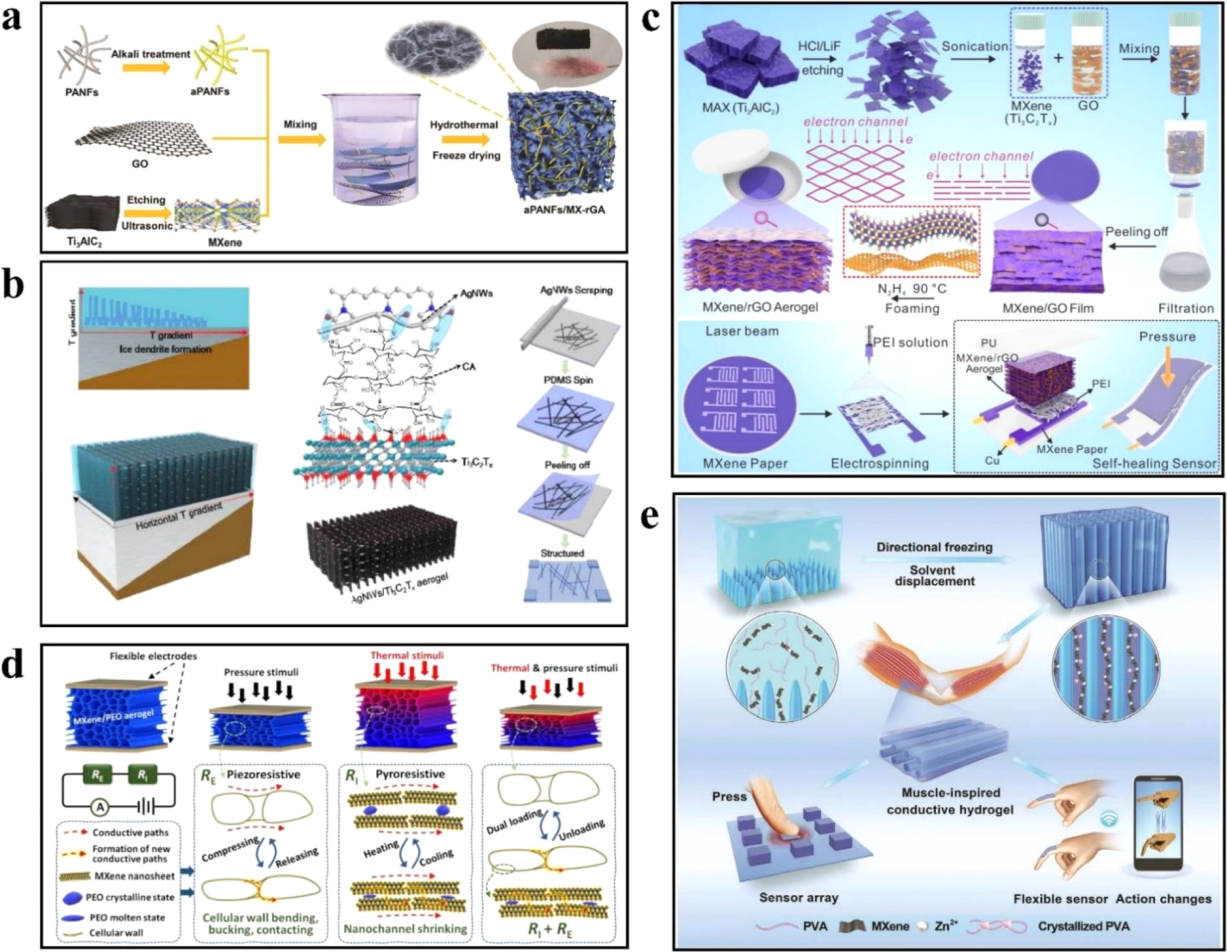
(a) Schematic diagram of the preparation process of aPANF/MX–rGA aerogel. Copyright© 2021, John Wiley and Sons. (b) Schematic diagram of preparation of AgNWs/Ti_3_C_2_T_*x*_ aerogel by directional freezing. Copyright© 2020, Royal Society of Chemistry. (c) Flexible pressure sensor with maximum electronic channel and self-healing prepared by gas foaming process. Copyright© 2023, American Chemical Society. (d) Schematic illustration of pyroresistive and piezoresistive mechanism for MXene/PEO aerogel. Copyright© 2022, Royal Society of Chemistry. (e) The synthesis procedures of PMZn–GL hydrogels and further applications in wearable flexible sensors and 3D sensor arrays. Copyright© 2021, John Wiley and Sons.

However, a single MXene material tends to stack easily, and its low aspect ratio and weak gel ability make it difficult to form a continuous porous structure. To address this issue, Niu *et al.*^50^ developed an alkali-based polyacrylonitrile nanofiber (aPANF/MX–rGA) aerogel with a three-dimensional interconnected porous structure. Polyacrylonitrile is prepared *via* electrospinning, and alkali treatment of the nanofibers enhances the interaction between polyacrylonitrile PAN nanofibers and graphene oxide (GO). The alkali-treated polyacrylonitrile nanofibers (aPANF) act as the scaffold for the aerogel network on the GO sheet, while the presence of MXene imparts excellent electrical conductivity to the aerogel. The resulting aerogel demonstrates high sensitivity (331 kPa^−1^ at 0–500 Pa and 126 kPa^−1^ at 500 Pa to 7.5 kPa), rapid response time (71 ms for load response, 15 ms for recovery response), and exceptional structural stability (17 000 compression cycles). This sensor exhibits the ability to detect weak signals from the body (Fig. 6a), such as pulse and heartbeat, with high sensitivity.

Bi *et al.*^51^ fabricated a composite aerogel of AgNWs/MXene through directional freezing. The directional freezing apparatus comprises two materials with significantly different thermal conductivities, aluminum and polyurethane, which generate horizontal and vertical temperature gradients during the freezing process. These temperature gradients facilitate the controlled growth of the AgNWs/Ti_3_C_2_T_*x*_ aerogel, resulting in a layered structure where the AgNWs intertwine between the Ti_3_C_2_T_*x*_ nanosheets to prevent stacking. The hydrogen bonding between AgNWs and Ti_3_C_2_T_*x*_ is achieved using calcium alginate (CA), formed by introducing sodium alginate (SA) and calcium chloride (CaCl_2_). The piezoresistive pressure sensor exhibits exceptional sensitivity (645.69 kPa^−1^), with a minimum detection limit of 1.25 Pa. It also offers a short response time of 60 ms and displays good bending performance within a bending angle range of 30° to 90°. Moreover, the sensor demonstrates high stability even after undergoing more than 1000 compression cycles (Fig. 6b). This sensor shows high sensitivity to low-pressure stimuli, enabling the detection of impact pressure generated by falling water droplets and collisions.

MXene's limited interlayer spacing and tendency to self-stack limit the changes in electronic channels under external pressure, thus hindering the exploitation of its excellent surface metal conductivity. Cheng *et al.*^23^ proposed a gas foaming method to construct MXene aerogel with adjustable layer spacing. Fig. 9a presents a flow diagram illustrating the preparation of MXene aerogel using hydrazine hydrate (N_2_H_4_) gas foaming. Fig. 9b and c depict the components and manufacturing process of the sensor. Firstly, an MXene paper-based interdigital electrode is prepared *via* laser engraving, and a layer of polyether imide (PEI) is electrospuned onto the interdigital electrode as a diaphragm. Finally, the components are assembled using self-healing polyurethane (PU). The interlayer porosity (54.4%) of the MXene aerogels prepared by gas foaming is significantly higher than that of the original structure (18.2%), resulting in high sensitivity (1799.5 kPa^−1^), a fast response time (11 ms), and good stability (>25 000 cycles). After experiencing mechanical damage, the carboxyl groups on the fractured surface are reconnected through hydrogen bonds. In the self-healing performance experiment, the sensor, which was cut in half by scissors, successfully reconnected and retained its initial sensing ability even after five recovery cycles while supporting a weight of 100 g (Fig. 6c).

Multi-parameter sensors can provide a more comprehensive and accurate description of the actual situation by monitoring multiple parameters simultaneously. Wu *et al.*^52^ presented a dual-sensing MXene aerogel capable of temperature and pressure measurements. By incorporating poly(ethylene oxide) (PEO) semi-crystalline polymers with varying molecular weights between MXene sheets, the PEO polymer undergoes changes in response to ambient temperature, thereby adjusting the distance between MXene sheets and altering the aerogel's resistivity for temperature sensing. Simultaneously, external pressure causes the lamellar structure of the MXene aerogel to contract or even bond, creating additional conductive pathways and modifying the aerogel's resistivity for pressure sensing (Fig. 6d). The aerogel demonstrates a pressure sensitivity of up to 777 kPa^−1^, with a detectable pressure limit of 0.05 Pa. Through the sensor's unique material selection and structural design, the thermopiezoresistive MXene/PEO aerogel enables the detection and differentiation of pressure and thermal stimuli within the physiological temperature range of the human body.

Hydrogel materials possess unique advantages in the realm of flexible sensing due to their high biocompatibility, strong adhesion, and remarkable self-healing ability. Enhancing the conductivity of hydrogel materials has become pivotal for their application as flexible sensors.

Feng *et al.*^53^ proposed a technique called directional freezing to fabricate MXene conductive hydrogels (PMZn) with anisotropy and low-temperature tolerance. In this method, MXene nanosheets were combined with polyvinyl alcohol (PVA) and zinc sulfate (ZnSO_4_) solutions and frozen at a low temperature. During the freezing process, the solvent solidified along the temperature gradient, squeezing other particles between the resulting ice-crystal columns. The ice crystal columns acted as orientation templates, creating an ordered internal orientation structure within the hydrogel. Subsequently, PMZn was immersed in glycerol to replace the solvent. Glycerol, forming hydrogen bonds with PVA and MXene nanosheets, replaced part of the water in PMZn, yielding PMZn–GL hydrogels. The hydrogel exhibited a tensile/compressive anisotropy ratio (defined as the ratio between the parallel and orthogonal directions) of approximately 2.2 and 1.36, respectively. The electrical conductivity of PMZn–GL, measured horizontally using an electrochemical workstation, reached 56 mS m^−1^, surpassing that of the orthogonal direction. These experiments showcased that the directed arrangement of the polymer network conferred excellent mechanical and electrical properties to the hydrogels. In the antifreeze test experiment, the horizontal tensile curve of PMZn–GL at −25 °C resembled that at room temperature, while the fracture strain of PMZn decreased at low temperatures. Differential scanning calorimetry (DSC) testing revealed a crystallization peak of −36.3 °C for PMZn–GL, significantly higher than that of PMZn (Fig. 6e). This finding indicated that PMZn–GL, obtained through glycerol replacement, exhibited enhanced resistance to low temperatures.

### 4.2 Ink printing

MXene, in comparison to other 2D materials, possesses a myriad of distinctive properties. These include metal-like electrical conductivity, superior dispersion, negative surface charge, and hydrophilicity. Such characteristics render it particularly well-suited as an ink for printing applications.^27^

**Fig. 7:**
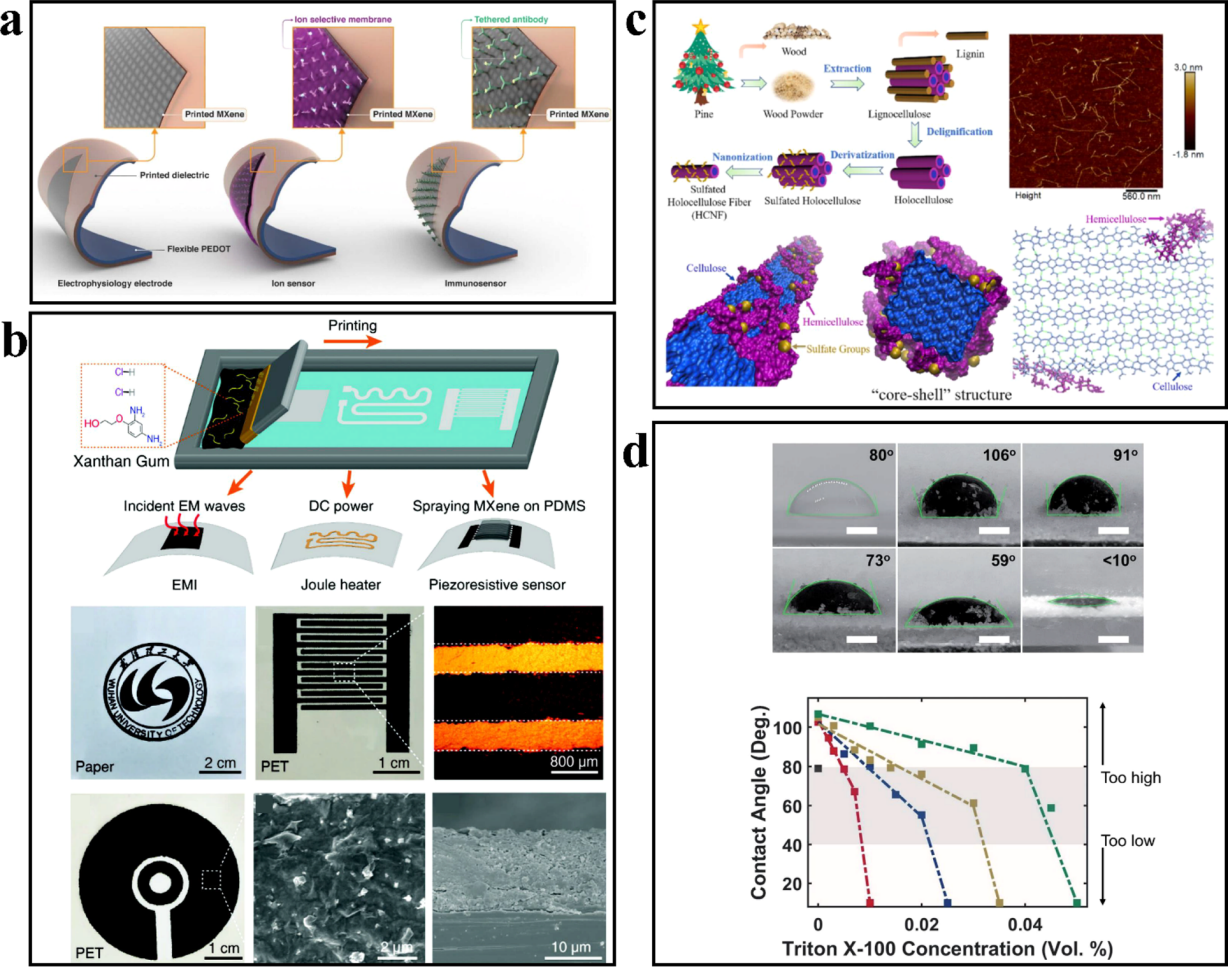
(a) Schematic illustration of three MXene electrodes printed on flexible self-standing PEDOT substrates. Copyright© 2020, IOP Publishing. (b) Screen-printing flexible MXene patterns for EMI, Joule heater and piezoresistive sensor devices. Copyright© 2022, John Wiley and Sons. (c) Schematic representations of sulfated HCNF with the “core–shell” structure. Copyright© 2021, American Chemical Society. (d) MXene inks for 3D printing vary in contact angle under different conditions. Copyright© 2022, Royal Society of Chemistry.

Inkjet printing provides a simple and cost-effective approach to fabricating wearable sensors. It allows for the deposition of desired patterns onto diverse substrates, including paper, thermoplastics, and glass. Saleh *et al.*^54^ employed the inkjet printing technique to deposit three layers of MXene ink onto poly(3,4-vinyldioxythiophene)–polystyrene sulfonic acid (PEDOT:PSS) to create the sensing film. The resulting film exhibited an electrical conductivity of 162.2 ± 24.2 S cm^−1^ and achieved a maximum bending radius of 2 cm during compression strain testing. The film demonstrated a consistent change in resistance even after 1000 bending cycles. Moreover, when exposed to air at room temperature for a period of 50 days, the resistance of the printed film only exhibited a slight increase (*R*/*R*_0_ = 1.19 ± 0.09), thereby highlighting its resistance to air-induced deterioration. This underscores the suitability of the printed film for applications involving exposure to air (Fig. 7a).

In comparison to inkjet printing, screen printing utilizes high-viscosity ink that is passed through a template screen and deposited onto the substrate, resulting in a higher deposition rate. Wu *et al.*^55^ developed a mixed ink of MXene and xanthan gum with adjustable viscosity and long-term stability, which was subsequently employed for screen printing the MXene/xanthan film. Regarding sensing performance, the MXene film fabricated using this approach exhibited a conductivity of 4.8 × 10^4^ S m^−1^, a rapid response time of 130 ms, and a stable detection capability under 30 kPa pressure. Furthermore, the MXene/xanthan film demonstrated exceptional electromagnetic shielding properties and Joule heating functionality. Its average electromagnetic interference shielding value reached 40.1 dB, and it achieved a heating rate of 20 °C S^−1^ in Joule heater applications, with a maximum steady-state temperature of 130.8 °C (Fig. 7b).

It possesses the function and internal structure similar to natural plant fibers while incorporating the advantages of cellulose fibers and nanocellulose, including high degradability, excellent biocompatibility, low density, high strength, large aspect ratio, and significant specific surface area. Chen *et al.*^56^ isolated lignocellulose by removing impurities from pine powder using acetone and subsequently extracted total cellulose from lignocellulose *via* a sodium chlorite (NaClO_2_)/acetic acid (CH_3_COOH) solution. The obtained cellulose was further modified with sulfamic acid and treated with a NaOH solution to obtain HCNF (hydroxylated cellulose nanofiber). MXene/HCNF (MH) inks were prepared by blending MXene and HCNF at varying mass fractions. The rapid decrease in the contact angle value of MH ink on cellulose paper demonstrates the excellent printability of MH ink. MH inks display typical non-Newtonian shear-thinning behavior, ensuring the maintenance of the structure and shape of the printed product, which exhibits consistent resistance signals over 101% strain cycles. The resistance of the printed product exhibits a continuous variation trend with changes in ambient temperature and humidity, and the strain sensing response remains almost constant after 3 months, indicating the stability of MH ink (Fig. 7c). 3D printing, also known as additive manufacturing (AM), is a rapid process for constructing 3D objects by printing layer by layer, offering high degrees of freedom and short lead times. Li *et al.*^57^ utilized adhesive jet (BJ) printing technology to combine polyvinyl alcohol (PVOH) with Ti_3_C_2_T_*x*_ material, which is easily soluble in water and possesses excellent biocompatibility. They successfully prepared a 3D/flexible MXene composite material measuring 4 cm × 1 cm × 1 mm. In the tensile test, the sample exhibited up to 250% deformation while maintaining stable conductivity at 50% deformation. The printed material demonstrated a linear sensitivity ranging from 0% to 80% strain, with a sensitivity value of 1.65 ± 0.16 over three repeated cycles (Fig. 7d).

### 4.3 Film/electronic skin

**Fig. 8:**
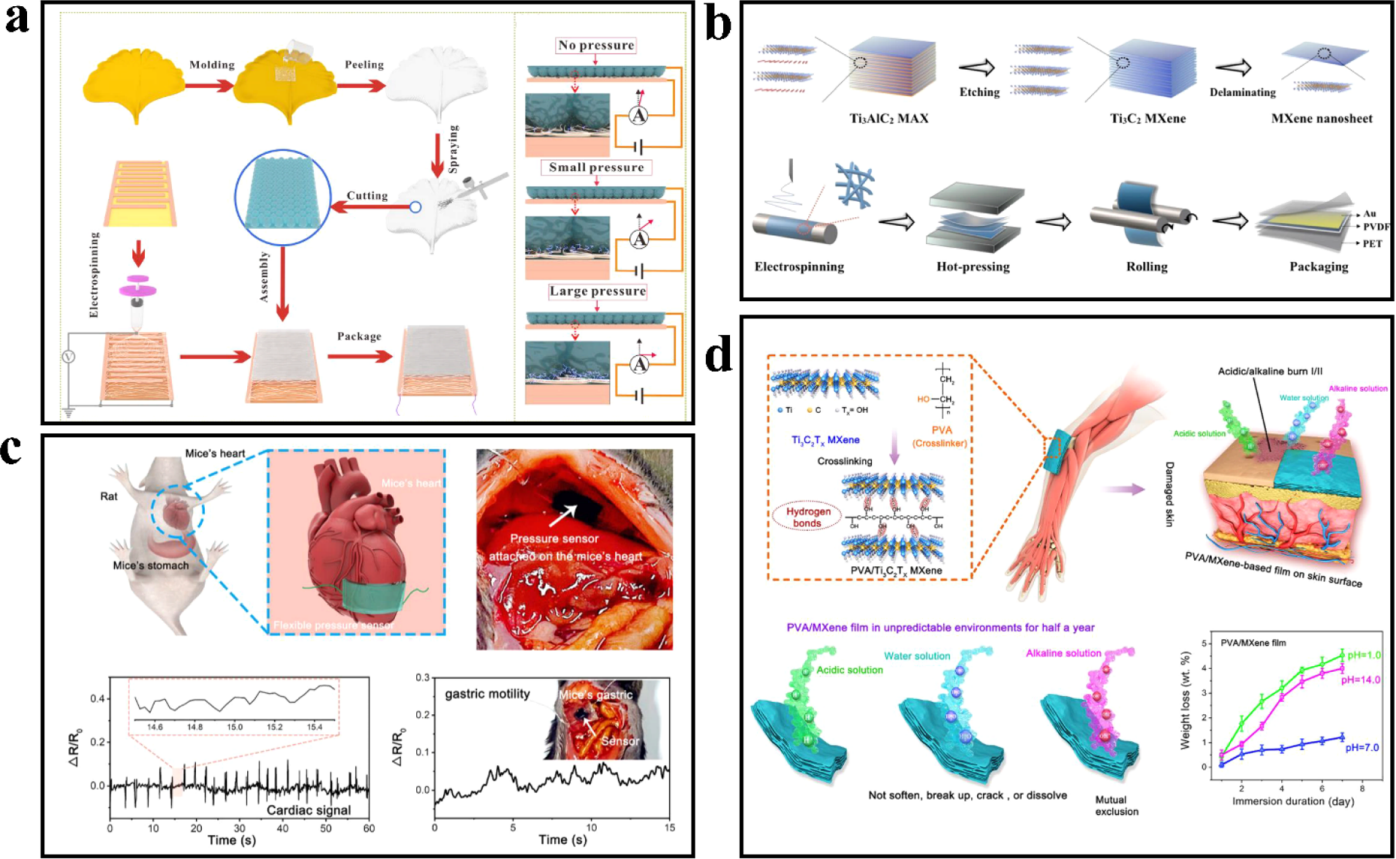
(a) Manufacturing technology of maple leaf bionic piezoresistive sensor. Copyright© 2022, Elsevier. (b) Structure diagram of piezoelectric PVDF hybrid film. Copyright© 2021, Elsevier. (c) Diagram of pressure sensors attached to the wall of the heart and stomach in mice. Copyright© 2021, Elsevier. (d) The schematic illustration of the skin-like PVA/MXene hybrid thin-film with crosslinked structures. Copyright© 2021, Elsevier.

The electronic skin, which mimics the perception function of human skin, is a novel type of thin, soft, and flexible film sensor. Yan *et al.*^58^ reported a piezoresistive sensing film with a bionic structure inspired by the ginkgo biloba leaf (Fig. 8a). The flexible sensor reproduced the microstructure of the ginkgo biloba leaf surface onto a polydimethylsiloxane (PDMS) film through molding. MXene nanosheets were then sprayed onto the film's surface to create a sensing layer. A layer of PVA fiber was inserted between the interdigital electrode and the film using electrospinning. The bionic thin film sensor exhibited a sensitivity of 403 kPa^−1^, a response time of 99.3 ms, a low detectable pressure limit (0.88 Pa), and demonstrated continuous stability over 12 000 load-unload cycles.

Similar to conductive enhancers such as graphene and carbon nanotubes, MXene can also induce the formation of piezoelectric phases, facilitate strong interfacial coupling effects, and generate piezoelectric responses. Zhao *et al.*^59^ proposed a highly sensitive piezoelectric sensor based on a mixed film of MXene and polyvinylidene fluoride (PVDF) by investigating the piezoelectric properties and sensitivity of MXene-modulated PVDF. The mixed film was fabricated through electrospinning, hot pressing, and roller pressing. To enhance the sensing performance of the sensor, Au electrodes were deposited on the film's surface using a corona polarization device, and the PVDF hybrid film was encapsulated with PET film. At a low loading level of 0.4 wt%, the piezoelectric coefficient *d*_33_ of the MXene/PVDF hybrid film reached a peak value of 43 pC N^−1^. Simultaneously, the addition of MXene nanosheets contributed to improving the mechanical properties of PVDF. The MXene/PVDF hybrid membrane sensor exhibited a voltage sensitivity of 0.0048 V N^−1^, which was twice that of the PVDF-based sensor (Fig. 8b).

The accurate recognition of physiological and physical signals is crucial for the performance of flexible sensors. However, improving the stability of flexible sensors in harsh environments remains a significant challenge. Zhao *et al.*^60^ developed a highly stable electronic skin, the MXene/PVA hybrid film, by synergistically binding strong hydrogen bonds between MXene and polyvinyl alcohol (PVA). The mixed membrane was fabricated by vacuum filtration of the MXene/PVA mixture through a cellulose membrane with a pore size of 0.22 nm. Through a 7 day soaking test in different solutions to assess its stability in various environments, the MXene/PVA mixed film exhibited weight loss of 1.2 ± 0.2 wt% in water, 4.0 ± 0.2 wt% in acid, and 4.5 ± 0.3 wt% in alkali solution, which was 22–83 times lower than that of the pure MXene membrane. Even after 24 hours of soaking, the mixed film maintained an elastic modulus of over 95%. In cell compatibility experiments, the survival rate of human umbilical vein endothelial cells after 7 days was 99.8 ± 0.9% (Fig. 8c and d), and the mixed membrane enabled stable *in vivo* heartbeat monitoring in anesthetized mice.

### 4.4 Fibrous fabric

**Fig. 9:**
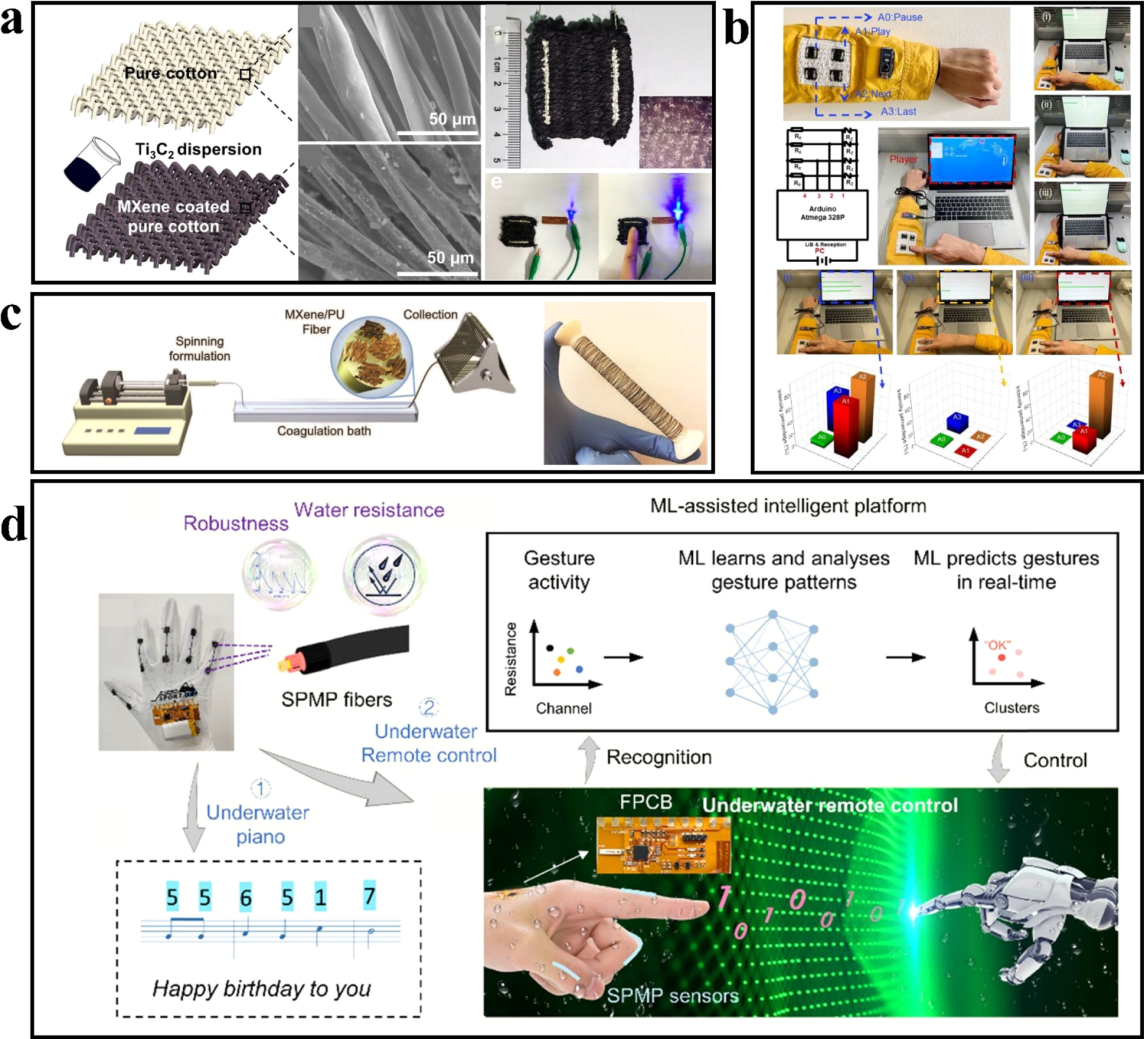
(a) Characterisation of the MXene-coated cotton pressure-sensing fabric.^54^ Copyright© 2020, American Chemical Society. (b) Proof-of-concept for MXene-coated cotton HMI systems. Copyright© 2020, American Chemical Society. (c) Schematic illustration of MXene/PU fiber spinning process. Copyright© 2020, John Wiley and Sons. (d) Design of the machine-learned sensory platform for underwater interactivities. Copyright© 2022, Elsevier.

The flexible device, comprised of fibers and flexible fabric, serves as a fundamental solution to address challenges associated with wearing comfort, wash resistance, and adherence to the human body. Simultaneously, it aligns with the principles of green environmental protection. The inherent roughness of cotton fibers promotes the adhesion of nanosheets and imparts excellent air permeability. Liu *et al.*^61^ achieved the fabrication of MXene cotton fabric by coating Ti_3_C_2_T_*x*_ nanosheets onto cotton fibers, resulting in strong bonding and consequently yielding a fabric with remarkable air permeability (972.2 mm s^−1^) and moisture permeability (227.92 g m^−2^). The sensor demonstrated exceptional performance, including high sensitivity (7.67), rapid response and recovery time (35 ms), outstanding stability (over 2000 cycles), and a wide sensing range (Fig. 9a and b).

Seyedin *et al.*^62^ fabricated MXene/polyurethane (PU) skin and PU core fibers through coaxial wet spinning. In comparison to non-coaxial composite fibers, these fibers exhibited enhanced stability when subjected to cyclic strains of varying magnitudes. The MXene/PU composite fiber demonstrated a strain sensing capacity of 152% and achieved a sensitivity (GF) of approximately 12 900. Knitted fabrics constructed with this fiber showcased a strain sensing ability of up to 200% and maintained exceptional stability even after undergoing 1000 cycles of stretch and release deformation (Fig. 9c). To enable tracking of diverse elbow movements, the MXene/PU fiber was woven into an elbow sheath using a knitting machine. Duan *et al.*^25^ devised a three-layer cored shell structure to create a smart fiber. The smart fiber, known as SPMP fiber, comprises four layers: a spandex core, a PVA layer, an MXene layer, and a PDMS layer. The SPMP fibers manifest a sensitivity (GF) of 10.3 within the strain range of 40% to 80%. When compared to unencapsulated fibers, the resistance variation of SPMP fibers remained within ±0.6% after being immersed in water, sweat, and saline solutions for 15 days, signifying exceptional washability and water resistance. To demonstrate the capabilities of the SPMP fiber, the researchers constructed a waterproof hybrid electronic system utilizing machine learning techniques. They deployed gloves as carriers for the flexible fibers, integrated fiber sensors, and processing centers onto a flexible printed circuit board (FPCB) using integrated packaging technology. Through this setup, they successfully accomplished underwater piano playing and recognized 20 gestures for remote-controlled robot hands with an accuracy rate of 98.1% (Fig. 9d).

We have summarized the preparation method and performance index for MXene-based sensing materials and presented them in Table 1.

**Table 1:** Performance data based on MXene pressure sensors

	Preparation method	Sensitivity/GF	Response time (ms)	Working range	Cyclic stability	Other properties	Ref.
Aerogel (aPANF/MX–rGA)	Freeze-dried	331 kPa^−1^	71	>7.5 kPa	17 000	—	50
Aerogel (AgNWs/Ti_3_C_2_T_*x*_)	Directional freezing	645.69 kPa^−1^	60	0–9 kPa	1000	—	51
Aerogel (rGO/MXene)	Gas-foaming	1799.5 kPa^−1^	11	>30 kPa	25 000	—	23
Aerogel (PEO/MXene)	Molecular intercalation	777 kPa^−1^	≈100	0–20 Pa	1000	Pressure and temperature dual sensing	52
Hydrogel (PMZn–GL)	Directional freezing	GF: 5.82	250	—	500	Frost resistance	53
Printing ink (PEDOT:PSS/MXene)	Inkjet-print	162 kPa^−1^	—	—	1000	Na^+^ ion selectivity	54
Printing ink (xanthan/MXene)	Screen printing	—	130	>30 kPa	1000	Electromagnetic shielding	55
Printing ink (HCNF/MXene)	Screen printing	—	—	—	10	Temperature/humidity sensing	56
Printing ink (PVOH/MXene)	3D printing	1.65 kPa^−1^	—	0–80% (tensile)	10	Energy storage	57
Mixed film (PDMS/PVA/MXene)	Moulding/spray	403 kPa^−1^	99.3	>20 kPa	12 000	—	58
Mixed film (PVDF/MXene)	Electrospinning/hot-pressing/rolling	0.048 V N^−1^	3.1	—	—	—	59
Mixed film (PVA/MXene)	Vacuum filtration	164.75 kPa^−1^	29	>100 kPa	7000	Cytocompatibility	60
Fibrous fabric (cotton fiber/MXene)	Coating	GF: 7.67	35	35% (tensile)	2000	—	61
Fibrous fabric (PU/MXene)	Coaxial wet-spinning process	—	—	200% (tensile)	1000	—	62
Fibrous fabric (SPMP)	Adhered/*situ* casting and curing	GF: 10.3	—	0–80% (tensile)	500	Water resistance	25

## 5 Summary and prospect

MXene materials exhibit excellent application potential in the realm of flexible pressure sensing owing to their remarkable electrical conductivity, mechanical properties, unique layered structure, and abundant functional groups on the surface. By integrating MXenes with other materials, a novel structural system can be developed to enhance the synergistic effects of MXene materials, leading to the creation of flexible sensors characterized by high sensitivity and a broad detection range. This paper presents an overview of research achievements in MXene-based flexible sensing across various domains, including hydrogel/aerogel, ink printing, paper-based film, and fiber fabric. The preparation strategies, sensing properties, and applications of these sensors in human body detection are also discussed. Despite the exceptional performance and potential displayed by MXene-based flexible pressure sensors, there still exist numerous challenges and issues that necessitate further exploration and resolution.

Assuredly, stability performance is crucial for flexible sensors, the easy oxidation of MXene material is still a problem to be solved, the sensor will inevitably oxidize during the working process, which will greatly reduce the stability and service life of the sensing device. Therefore how to enhance the service life of MXene-based flexible sensors is an issue worthy of further research. Secondly, among the MXene-based flexible sensors reported now, due to the limitations of material properties and manufacturing processes, they have not yet been able to meet the demands of some specific applications, such as in high temperature, high humidity, or strong corrosive environments, the performance of MXene flexible sensors will be affected, which restricts the application in these environments. Packaging plays a critical role in the functionality of flexible sensors, particularly in wearable sensor applications. Appropriate packaging techniques allow subjects to wear the device for extended durations while minimizing signal drift and noise. Hence, it becomes essential to identify suitable packaging methods that enable the acquisition of more accurate human data and enhance wearer comfort. Finally, the mechanical forces applied to sensors typically involve a combination of pressure, tension, shear, and torsion forces. Decoupling these mixed forces becomes particularly crucial for applications such as gesture recognition, robot control, and prosthetics. To achieve this, sophisticated structural designs are needed to differentiate between different types of mechanical stimuli and assign them to the corresponding sensors. This enables independent detection of deformations caused by distinct forces.

## Conflicts of interest

There are no conflicts to declare.

## Supplementary Material
